# The Peroxisomal Targeting Signal 3 (PTS3) of the Budding Yeast Acyl-CoA Oxidase Is a Signal Patch

**DOI:** 10.3389/fcell.2020.00198

**Published:** 2020-03-27

**Authors:** Błażej Kempiński, Anna Chełstowska, Jarosław Poznański, Kamil Król, Łukasz Rymer, Zuzanna Frydzińska, Wolfgang Girzalsky, Adrianna Skoneczna, Ralf Erdmann, Marek Skoneczny

**Affiliations:** ^1^Institute of Biochemistry and Biophysics, Polish Academy of Sciences, Warsaw, Poland; ^2^Medizinische Fakultät, Biochemie und Pathobiochemie/Systembiochemie, Ruhr-Universität Bochum, Bochum, Germany

**Keywords:** PTS3 signal, *in vivo* import, fluorescence microscopy, two-hybrid screen, 3D modeling

## Abstract

The specificity of import of peroxisomal matrix proteins is dependent on the targeting signals encoded within their amino acid sequences. Two known import signals, peroxisomal targeting signal 1 (PTS1), positioned at the C-termini and PTS2 located close to N-termini of these proteins are recognized by the Pex5p and Pex7p receptors, respectively. However, in several yeast species, including *Saccharomyces cerevisiae*, proteins exist that are efficiently imported into peroxisomes despite having neither PTS1 nor PTS2 and for which no other import signal has been determined. An example of such a protein is *S. cerevisiae* acyl-CoA oxidase (AOx) encoded by the *POX1* gene. While it is known that its import is driven by its interaction with the N-terminal segment of Pex5p, which is separate from its C-terminal PTS1-recognizing tetratricopeptide domain, to date, no AOx polypeptide region has been implicated as critical for this interaction, and thus would constitute the long-sought PTS3 signal. Using random mutagenesis combined with a two-hybrid screen, we identified single amino acid residues within the AOx polypeptide that are crucial for this interaction and for the peroxisomal import of this protein. Interestingly, while scattered throughout the primary sequence, these amino acids come close to each other within two domains of the folded AOx. Although the role of one or both of these regions as the PTS3 signal is not finally proven, our data indicate that the signal guiding AOx into peroxisomal matrix is not a linear sequence but a signal patch.

## Introduction

The mechanisms by which the majority of proteins are imported into the peroxisomal lumen are well known and are based on the recognition of short stretches of amino acids located either at the C-terminus (peroxisomal targeting signal 1, PTS1) or near the N-terminus (PTS2) of their polypeptides by receptors that recognize these signals, Pex5p or Pex7p, respectively (Girzalsky et al., 2010). For three decades after the discovery of these signals, our knowledge on these mechanisms has been constantly enriched with the details, which resulted in the formulation of comprehensive models of PTS1- and PTS2-dependent import (Walter and Erdmann, 2019). However, not all peroxisomal matrix proteins have recognizable PTS1 or PTS2 signals; therefore, alternative mechanisms of their import into this organelle must exist. One possibility may be the piggy-back mechanism, which is characteristic for peroxisomes because of their unique ability to import native, fully folded and co-factor-bound proteins even in complex with other proteins. Therefore, a protein devoid of PTS signal may enter peroxisomes by being bound to another protein that has one (Glover et al., 1994). The commonness of this import method remains to be determined; however, very few examples of naturally occurring piggy-back import have been documented thus far (Yang et al., 2001; Titorenko et al., 2002; Effelsberg et al., 2015). Another mechanism appears to be employed by acyl-CoA oxidase (AOx) in *Saccharomyces cerevisiae*. Few studies have been devoted to this protein, and our understanding of its import mechanism is limited. Nevertheless, these studies revealed that *S. cerevisiae* AOx is recognized by the Pex5p receptor, the same that functions in the PTS1-dependent import, but the recognition domain for AOx lies within the N-terminal part of Pex5p, separate from its C-terminal tetratricopeptide domain (TPR) that binds the PTS1 signal (Skoneczny and Lazarow, 1998). The region between amino acid residues 250 and 270 with the Ile264 plays a crucial role in AOx recognition and import (Klein et al., 2002). This surprising finding portrays Pex5p as a dual-domain, dual-function peroxisomal import receptor and suggests that the mechanism of AOx import may converge with the well-established PTS1-dependent import route. Interestingly, the N-terminal half of Pex5p is also important for the recognition and peroxisomal import of carnitine acetyltransferase (Cat2p), which possesses a functional PTS1 signal in the C-terminus that can be abrogated without noticeable consequences to localization of this protein (Klein et al., 2002). Moreover, we recently demonstrated that two other peroxisomal proteins, (1) the multifunctional enzyme of the peroxisomal fatty acid beta-oxidation pathway that comprises the 3-hydroxyacyl-CoA dehydrogenase and enoyl-CoA hydratase (Fox2p) and (2) the peroxisomal catalase A, both containing the PTS1 signals at their C-termini, are also partially dependent on this side branch of the Pex5p-dependent import route (Rymer et al., 2018). It remains an open question whether the list of *S. cerevisiae* proteins interacting with the N-terminal half of the Pex5p *en route* to peroxisomes is complete. The participation of both functional parts of Pex5p in the import of the PTS1-containing peroxisomal proteins also remains to be determined. While the very existence of this side branch of the Pex5p-dependent import mechanism employed by AOx is well established, to date, we do not know the corresponding module within the AOx molecule that is recognized by the N-terminal region of the Pex5p receptor.

In this study, we used random mutagenesis of the AOx-encoding gene to identify AOx amino acid residues that are involved in its interaction with the N-terminal half of Pex5p and its import to peroxisomes. Surprisingly, the amino acid substitutions that affected AOx-Pex5p interaction and AOx import were scattered widely throughout the central part of the AOx polypeptide, comprising approximately half of the whole protein. However, in the model of the AOx tertiary structure, they cluster together into two small regions. The *in vivo* import tests demonstrated the importance of these amino acid residues for the import of AOx into the peroxisomal matrix, indicating that they form a signal patch that functions as novel peroxisomal targeting sequence.

## Materials and Methods

The XL1-Blue (Stratagene) bacterial strain was used for plasmid construction and propagation. PJ69-4A (James et al., 1996) was used as a host for two-hybrid screens and tests. BYpex5Δ,pox1Δ, a double-deletion derivative of BY4741 (Open Biosystems, Huntsville, AL, United States), was used for tests of efficiency of AOx import *in vivo*. The yeast strains were grown at 28°C either in rich YPD medium or in synthetic complete (SC) selective medium with 2% glucose or with a mixture of 0.1% glucose, 2% ethanol, 0.1% oleic acid, and 0.25% Tween 80 to induce peroxisome proliferation.

The plasmids that were used in this study are listed in Supplementary Table S1.

### Two-Hybrid Screen for the Amino Acid Substitutions in the AOx Polypeptide That Affect Its Interaction With the N-Terminal Region of the Pex5p Receptor

*POX1* ORF was randomly mutagenized by mutagenic PCR using a GeneMorph II random mutagenesis kit (Agilent, Santa Clara, CA, United States), according to the manufacturer’s protocol, to obtain an average single mutation per 1000 bp. Two hybrid libraries were made directly in yeast cells using gap repair method by transforming PCR products together with linearized pGBT9-AOx two-hybrid plasmid into a PJ69-4A strain previously transformed with the pGAD-Pex5p^136–292^ plasmid. Transformant clones displaying affected growth on the medium without adenine were isolated; plasmid DNA was prepared and amplified from them; and the inserts including the *POX1* ORF were sequenced.

### Quantification of the Two-Hybrid Interactions Between AOx and the N-Terminal Region of Pex5p

pGBT-AOx plasmids bearing mutations within the *POX1* ORF, selected in a two-hybrid screen, were transformed into the PJ69-4A strain together with the pGAD-Pex5p^136–292^ plasmid. The two-hybrid interaction displayed by the transformant clones was quantified by a plate test of growth in medium without adenine supplementation and by measuring the intracellular β-galactosidase activity according to the method published by Guarente (1983).

### Modeling the Structure of ScAOx

Yasara Structure v. 19.5.5^1^ protein modeling software was used to model the structure of *S. cerevisiae* AOx based on its homology to AOx proteins in other organisms, for which crystallographic structures are known. The structures of acyl-coenzyme A oxidase in *Yarrowia lipolytica* (5YS9;5Y9D), *Rattus norvegicus* (1IS2;2DDH), and *Arabidopsis thaliana* (1W07) were automatically selected as the best templates according to the combination of the Blast *E*-value, sequence coverage, and structure quality. For each template, up to five alternate alignments with the target sequence were used, and up to 50 different conformations were tested for each modeled loop. The resulting models were evaluated according to structural quality (dihedral distribution, backbone, and side-chain packing). The model with the highest score was built based on the 5YS9 structure; however, the chimerical model, in which some parts were adopted from other highly scored models (2DDH, 1W07), scored even higher (see Supplementary Video S1 for the regions improved by this procedure). The final model contained the FAD molecule, the location of which was taken directly from the template 5YS9 structure.

### *In vivo* Import Assay of AOx Bearing Amino Acid Substitutions Identified in a Two-Hybrid Screen

Mutations within the *POX1* ORF of the pGBT-AOx two-hybrid plasmid were transferred into the AOx-GFP plasmid (Rymer et al., 2018). The plasmids were transformed into the BYpex5Δ,pox1Δ strain together with the mRFP-SKL plasmid encoding a synthetic peroxisomal marker (Rymer et al., 2018) and the pRS413-Pex5WT or pRS413-Pex5IK plasmids encoding either the wild-type or the I264K-substituted version of the Pex5p receptor. Transformant cells were grown at 28°C in SC selective medium without histidine, leucine and uracil with Tween 80/oleate as a carbon source for 44 h to induce peroxisome proliferation. Cell images were acquired with Zeiss Axio Imager.M2 microscope with AxioCam MRc5 camera and AxioVision release 4.8 software, under 1000x magnification with EC Plan-NEOFLUAR 100x objective. 38HE green filter and 63HE red filter were used for fluorescence imaging. At least 100 cells in three biological replicates for each combination of plasmids were categorized according to AOx-GFP protein localization as displaying pure peroxisomal, mixed peroxisomal/cytosolic or pure cytosolic localization. The average of the results data was determined, the standard deviation was calculated and the significance was determined with Student’s *t*-tests.

See Supplementary Material for the expanded version of the “Materials and Methods” section.

## Results

For the search of import signal(s) that direct *S. cerevisiae* AOx to the peroxisomal matrix, we used a two-hybrid screen combined with random mutagenesis of the *POX1* ORF. In this approach, the library of mutated versions of the *POX1* ORF that were fused in frame to sequence encoding the Gal4 DNA-binding domain in the pGBT9 vector was screened with the fragment of the *PEX5* gene between 136 and 292 codon fused in frame to the sequence encoding the Gal4-activating domain in the pGAD424 vector (see Supplementary Material for the detailed description of the two-hybrid screen strategy). Previously (Skoneczny and Lazarow, 1998), we found that this relatively small fragment of Pex5p, outside the PTS1-recognizing TPR domain, did robustly interact with AOx in the two-hybrid system and that both polypeptides co-purified in metal affinity chromatography. Therefore, we applied this experimental setup to identify single amino acid residues that are involved in the AOx interaction with the same region of Pex5p. To this end, AOx was randomly mutagenized and resulting mutants were tested for their interaction with Pex5p. The first round of screening resulted in a list of more than one hundred clones, which were retested for the two-hybrid interaction to get rid of false positives. The resulting twelve mutant clones encoding BD-AOx displayed an affected interaction with AD-Pex5p^136–292^ reproducibly. Initially, our intention was to isolate the clones conferring loss of interaction, but we noticed that some of the clones conferred mere weakening of the interaction, and some, to our surprise, conferred strengthening of interaction. We decided to include these clones into our clone set.

### The Amino Acid Residues Participating in the AOx Interaction With Its Receptor Are Concentrated Within Several Distinct Regions in the Middle of the AOx Polypeptide

**FIGURE 1:**
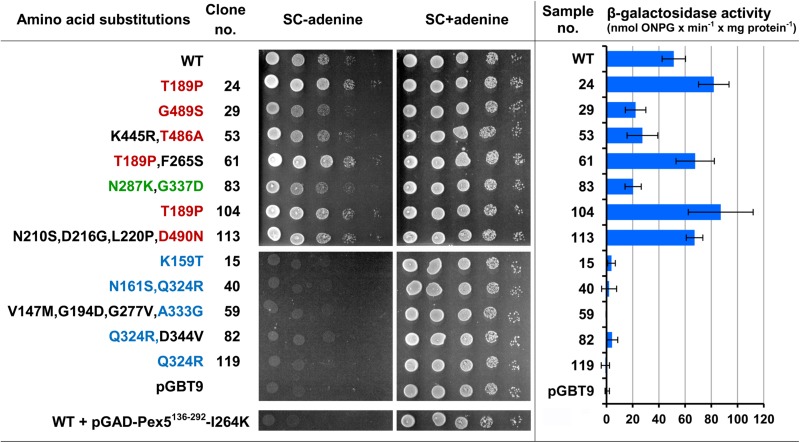
Test of two-hybrid interaction between the mutated AOx and the Pex5p fragment comprising amino acids 136 to 292 that contains the AOx binding site. The left panel shows the growth of PJ69-4A cells transformed with pGAD-Pex5^136– 292^ and with either pGBT-AOxWT or with its derivatives encoding mutated BD-AOx fusions or with pGBT9 empty vector as a control. For comparison, the growth of PJ69-4A cells transformed with pGAD-Pex5^136– 292^-I264K, encoding the mutated two hybrid construct (see the main text for more details regarding the I264K amino acid substitution), and with pGBT-AOxWT, is also presented. Transformant cells cultured overnight were serially diluted 10×, spotted onto a solid SC selective medium without adenine. and the same medium with adenine as a control of spotting uniformity and incubated for 2 days at 28°C. The right panel shows the level of β-galactosidase expressed due to the two-hybrid interaction in the cells transformed with the same plasmid combinations. The results show the average of the measurements taken for three independent transformant clones after subtraction of the β-galactosidase background activity levels determined in cells transformed with the same set of pGBT9-derivative plasmids together with the empty pGAD424 vector. Error bars represent the standard deviation. Clone and sample numbers refer to the number of clones isolated in the first round of the screen. Amino acid substitutions that most likely are important for the AOX-Pex5p interaction in upper and lower group of clones are highlighted in red or blue, respectively.

Figure 1 shows the results of two-hybrid interaction assays for all twelve clones as performed with two methods: determining the growth ability on solid medium lacking adenine and measuring β-galactosidase activity (see Supplementary Material for more details). Clones conferring weakened or strengthened interaction are grouped in the upper part of the figure, whereas those with no interaction are grouped in the lower part of the figure. Figure 1 also shows the amino acid substitutions identified in these clones. One can notice that they are not randomly distributed throughout the AOx polypeptide. Moreover, some substitutions occurred more than once at the same or neighboring positions, which suggested that the screen was saturated. In the majority of clones at least one such substitution was found. In the lower group of clones single substitutions, namely, K159T (clone 15) and Q324R (clone 119), were sufficient to abolish the interaction. The same effect was found for a combination of Q324R with N161S (clone 40) or with D344V (clone 82), suggesting the crucial role of Q324 residue and the K159-N161 region of AOx in its interaction with Pex5p. The correlation between the positions of substituted residues on the AOx polypeptide and the strength of its interaction with Pex5p is less clear-cut in the upper group of clones showing weakened or strengthened interactions, but it is clear that an amino acid residue substitution T189P (clones 24 and 104) alone or in combination with F265S (clone 61) strengthened the interaction and that this interaction was weakly affected by substitutions at positions 486–490. Clearly, the amino acid residues in the AOx polypeptide that seem to be important for its interaction with Pex5p are located within four small, distinct regions: around K159, T189, Q324, and D490. The interaction was also abolished by the combination of V147M, G194D, G277V, and A333G substitutions (clone 59), although it is not clear, which of these residues actually participate in the interaction. Residue A333, located near Q324 seems to be a good candidate, but on the other hand, residue G194 is located close to T189. The only exception is clone 83. Both substitutions: N287K and G337D do not seem to fit the emerging picture outlined above.

To distinguish the recurring substitutions from the remaining ones, they were marked in Figure 1 with different colors. Those around T189 and D490, occurring mostly in the upper group of clones were highlighted with the red color and those around K159 and Q324 from the lower group, with blue color. As it will become apparent in the next subchapter, there is a correlation between the two-hybrid interaction phenotype and the localization of these residues within folded AOx molecule. The substitutions in exceptional clone 83 were marked with green color.

### The Amino Acid Residues Participating in the AOx Interaction With Pex5p Form Two Distinct Regions on the Surface of the AOx Monomer

**FIGURE 2:**
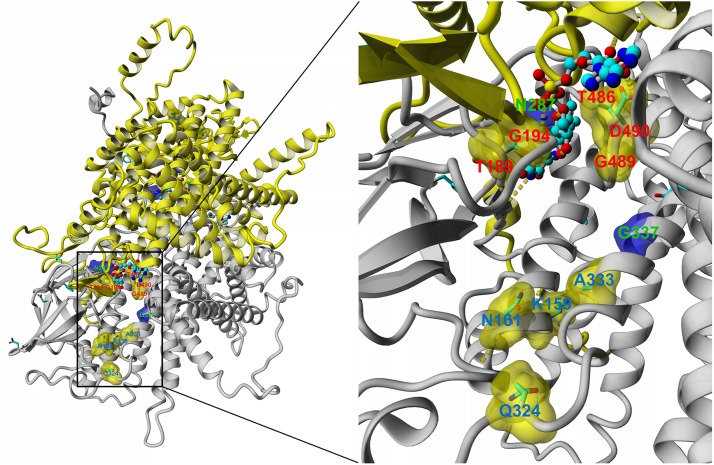
Left panel: The model of the *S. cerevisiae* AOx dimer generated with Yasara modeling software on the template of the known crystal structure of *Y. lipolytica* ACOX1. Amino acid residues highlighted with red, green or blue color in Figure 1 are labeled here with a capital letter and the residue number of the same color. For better visibility green-labeled N287 and G337 are put on the blue background. The side chains of all amino acid residues that were found, as substituted in our screen, are shown. Black rectangle marks the regions of interest enlarged on the right side of the figure. Right panel: The enlargement of both regions of interest showing the important areas for interaction with Pex5p. See also Supplementary Videos S2, S3 for the animated, rotating model of AOx.

The observation that the amino acid residues whose substitution affects the interaction of AOx with the Pex5p receptor are localized in several hot spots in the AOx polypeptide strongly suggested that these residues participate in this interaction. Therefore, we attempted to localize them within the natively folded AOx protein. To this end, we generated a structural model of *S. cerevisiae* AOx. While the structure of this protein has not yet been determined experimentally, the crystallographic structures of AOx proteins from many organisms belonging to other systematic groups are known. Importantly, two isoenzymes of *Y. lipolytica* AOx, ACOX1, and ACOX3, have been crystallized recently, and their structures were determined at 2.5 Å resolution (Kim and Kim, 2018a, b). The budding yeast AOx shares 36% identity and 58% similarity (PAM250 similarity matrix) with both of these crystallized *Y. lipolytica* ACOX proteins. This level of homology (Blast *E*-value below 10^–100^, coverage of ∼90% for *Y. lipolytica* proteins) enabled the modeling of yeast AOx, based mostly on the template of YlACOX1 and YlACOX3 crystallographic structures, at a true atomic level of precision, particularly for the residues located in the core region of the protein. Yasara Structure package v. 19.5.5 was used for this purpose (see “Materials and Methods” section for more details). The resulting model of the whole ScAOx molecule dimer and the enlargement of the region of interest is presented in Figure 2. The substituted residues that are marked red, green or blue in Figure 1 are highlighted with the same color. See also Supplementary Videos S2, S3 for an animated view of the whole AOx molecule and its fragment, respectively. Interestingly, residues K159, N161, Q324, and A333G were clustered together into one domain on the surface of the AOx model, whereas residues T189, G194, T486, G489S, and D490N were found within the FAD-binding pocket. Notably, as explained above, while the substitution of residues belonging to the surface cluster abolished the AOx interaction with Pex5p, the substitution of the residues located in the FAD-binding pocket had only a limited effect on the interaction or even increased the strength of the interaction. Residues N287 and G337 substituted in clone 83 are present inside the modeled AOx molecule so the effect of these substitutions most likely results from the distortion of the AOx structure that indirectly influences its interaction with Pex5p receptor.

### The Amino Acid Residues Substituted in the AOx Polypeptide Are Important for Efficient Peroxisomal Import

**FIGURE 3:**
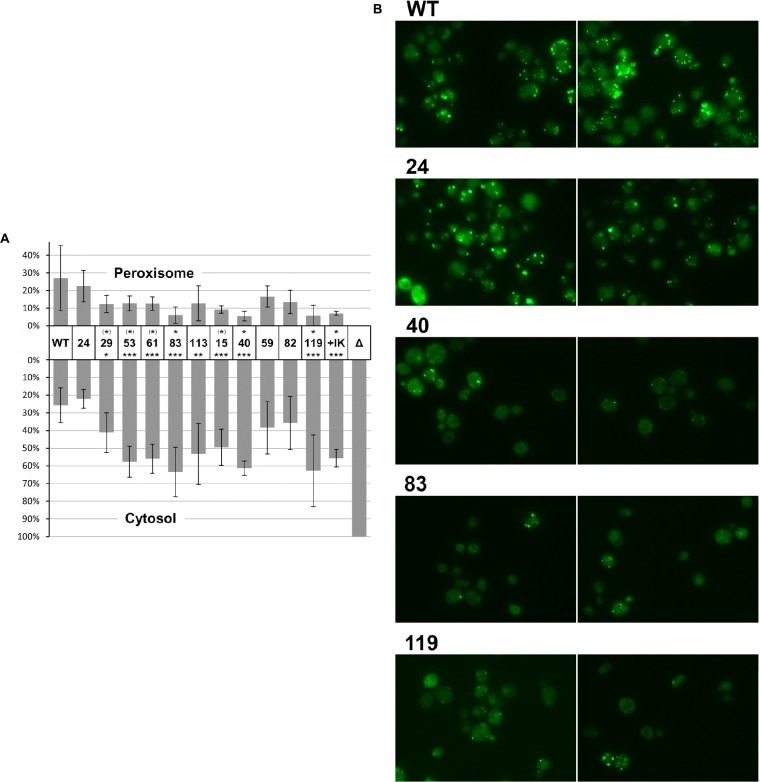
**(A)** Quantification of the *in vivo* import of AOx C-terminally tagged with GFP, bearing the amino acid substitutions identified in the two-hybrid screen. Individual columns on the graph are labeled with numbers referring to the original two-hybrid clones and are ordered as in Figure 1. Clone no. 104 was omitted because it had the same mutation as clone no. 24. For comparison, the data for the import of wild-type AOx-GFP either in the presence of wild-type (WT) or in the presence of I264K-substituted (+IK) Pex5p or in the absence of Pex5p (Δ) are shown. Plasmids encoding wild-type and mutated AOx-GFP proteins are described in Supplementary Material. Cells were categorized as displaying peroxisomal, cytosolic or mixed peroxisomal/cytosolic localization of AOx-GFP and the number of cells is expressed as a percent of total number of cells counted. The differences in the percent of cells belonging to mixed category between the mutated AOx-GFP clones and the wild-type AOx-GFP clone were not statistically significant therefore, for clarity, only the data for peroxisomal and cytosolic localization are shown in the graph. The complete data are shown in Supplementary Figure S1. Error bars represent the standard deviation. Statistical significance was determined for peroxisomal and cytosolic localization data (upper and lower row of stars, respectively), relative to wild-type AOx-GFP (WT column) and was calculated with Student’s *t*-test: ***P < 0.005, **P < 0.01, *P < 0.05, (*)*P* < 0.1. **(B)** The representative images of cells expressing the wild-type AOx-GFP and those with substitutions found in clones 24, 40, 83, and 119.

Amino acid residues participating in the AOx interaction with the N-terminal region of Pex5p are also likely to be important for AOx peroxisomal import. To test this assumption, the mutations in the clones that we selected in the two-hybrid screen were introduced into the gene encoding the full-length AOx C-terminally tagged with GFP, to determine their influence on the import of AOx into peroxisomes. The plasmids constructed this way and the plasmid bearing no mutations were separately transformed into BYpex5Δ,pox1Δ containing the mRFP-SKL plasmid encoding a peroxisomal fluorescent marker and pRS-Pex5pWT encoding the wild-type Pex5p receptor. For comparison, the strains bearing plasmids encoding the original AOX-GFP protein and pRS-Pex5pI264K encoding the mutated Pex5p receptor or not expressing Pex5p receptor at all were included in the analysis. Transformed cells were grown in oleate-containing medium to promote the proliferation of peroxisomes and examined by fluorescence microscopy. The cells were categorized according to the extent to which AOx-GFP was imported into the cells: perfect peroxisomal import, mixed peroxisomal/cytosolic localization and only cytosolic localization. The results of this experiment are shown in Figure 3A. Since the fraction of cells showing mixed peroxisomal/cytosolic localization did not differ strongly between the wild-type and the mutated clones this category of cells was omitted from this graph. The complete data are shown in Supplementary Figure S1. In most cases, the analyzed amino acid substitutions affected the import of AOx-GFP, and the differences between the cells expressing the mutated and the native versions of AOx-GFP, in terms of the percent of cells belonging to individual categories, were mostly statistically significant for cytosolic distribution and to a lesser extent for peroxisomal localization of green fluorescence. The strongest import defect was seen for AOx-GFP with substitutions pertaining to clones 40, 83 and 119. The notable exception was T189P substitution (clone 24), which curiously made the two-hybrid AOx-Pex5p interaction stronger than that conferred by the wild-type AOx. In the *in vivo* import test, this substitution did not affect the import of AOx into peroxisomes. The representative images of cells expressing the wild-type AOx-GFP and those with substitutions found in clones 24, 40, 83, and 119 are shown in Figure 3B.

For comparison, Figure 3A shows also the quantitative data for the *in vivo* import of the wild-type AOx polypeptide fused to GFP in the presence of the mutated Pex5p receptor, in which isoleucine at position 264 was substituted with lysine (IK labeled column). As previously found, substituting this amino acid with lysine (Rymer et al., 2018) or with threonine (Klein et al., 2002) compromised PTS3-dependent import and abolished Pex5p interaction with AOx and Cat2p without affecting the Pex5p interaction with the peroxisomal translocon proteins Pex13p and Pex14p. As shown in Figure 3A, the effect of most of the AOx mutations identified through our screen was generally similar to the effect of the I264K substitution in Pex5p.

## Discussion

The mechanism by which AOx is imported into *S. cerevisiae* peroxisomes has remained a mystery for decades. In contrast to our increasing understanding of the evolutionary conserved peroxisomal targeting of protein via PTS1- or PTS2-dependent pathways, our knowledge on the mechanisms of peroxisomal import of AOx and related proteins is still scarce. Based on the few known facts accumulated for peroxisomal targeting of these proteins we may conjecture that the corresponding mechanisms are distinct.

The import of budding yeast AOx into peroxisomes is dependent on the known PTS1 receptor Pex5p, but does not involve its PTS1-recognizing TPR domains or the C-terminal amino acids of AOx. Instead, AOx binds to the N-terminal part of Pex5p (Skoneczny and Lazarow, 1998; Klein et al., 2002), which led us to propose the hypothesis that Pex5p is a dual-function dual-domain receptor. Similar Pex5-dependent PTS1-independent import was demonstrated for *Hansenula polymorpha* alcohol oxidase (Gunkel et al., 2004). On the other hand, *Y. lipolytica* ACOX import is driven by its interaction with Pex20p, known as a coreceptor in the PTS2-dependent pathway, but it does not rely on this pathway or on the PTS1/Pex5p mechanism (Chang and Rachubinski, 2019). Nevertheless, regardless of the mechanism of import for any of these proteins, a specific region within their polypeptide chain must be recognized by the peroxisomal receptor that is specific for their import. If not present at the N- or C-terminus, then this region must be located somewhere internally, and in few reports, such a location was suggested for *Candida tropicalis* AOx (Small et al., 1988) and for *S. cerevisiae* catalase A (Kragler et al., 1993). The same is true for *S. cerevisiae* AOx, which is imported, as we have shown recently, independently of PTS1 and PTS2 (Rymer et al., 2018). However, in none of the known cases of these alternative import mechanisms the region responsible for the peroxisomal targeting was identified with greater accuracy. This paper is, to our knowledge, the first to demonstrate the design of such an internal region within *S. cerevisiae* AOx with precision to individual amino acid residues. Using random mutagenesis of the *POX1* ORF combined with the two-hybrid screen, we identified residues whose substitution abolishes the AOx interaction with Pex5p and makes AOx peroxisomal import less efficient. Remarkably, these amino acids, namely K159, N161, Q324, and perhaps also A333, cluster in a putative Pex5p-interacting domain that is, as one could expect, located at the surface of the deduced model of AOx. Thus the binding interface is formed by a cluster of several amino acid residues from two regions of the AOx polypeptide separated by 160 amino acids, which are in close proximity in the natively folded protein.

Moreover, we identified the FAD-binding pocket as another region, where amino acid substitutions affect AOx interactions with Pex5p and its peroxisomal import, albeit to a lesser extent. Notably the FAD-binding pocket, as well as residues substituted in clone 83, are buried inside the protein, as shown in the model of AOx structure (see Figure 2 and Supplementary Videos 2, 3).

The two sets of data: two-hybrid interaction of AOx with Pex5p (see Figure 1) and AOx-GFP import *in vivo* (see Figure 3) correlate with each other well but not perfectly. This lack of full correlation may be explained by the difference between these two experimental approaches. The two-hybrid interaction was studied between AOx and the 136–292 aa fragment of Pex5p receptor, whereas *in vivo* AOx-GFP import was studied in the presence of full-length Pex5p. This suggests that the import of AOx depends also on interaction of another AOx region, additional to K159, N161, Q324 domain, with the region of Pex5p outside its 136–292 domain. That is why we see some import of AOx-GFP proteins with substitutions preventing two-hybrid interaction (compare lower part of Figure 1 with the right side of Figure 3A). Notably, the influence of substitutions pertaining to clones 40 and 119 on two-hybrid interaction and *in vivo* import of AOx are similar to the effect of I264K substitution in Pex5p on the interaction and *in vivo* import of wild-type AOx (compare the bottom of Figure 1 and the columns labeled 40, 119, and +IK in Figure 3A).

The postulated additional Pex5p-interacting region in AOx may be the FAD-binding pocket as revealed by our two-hybrid screen. Alternatively, it may be located elsewhere and the substitutions within FAD pocket, as well as the substitutions N287K, G337D found in clone 83, may simply distort the folding of AOx. This would affect the *in vivo* import of AOx-GFP. It could also weaken or strengthen the interaction of AOx K159, N161, Q324 domain with 136–292 region of Pex5p receptor (compare upper part of Figure 1 with the left side of Figure 3A).

The inconsistency regarding clones 82 and 119 is more difficult to explain. Perhaps an additional substitution in clone 82 of D344 residue, buried inside the AOx molecule, distorts its structure in a certain way and thereby alleviates the *in vivo* import defect caused by Q324R substitution (compare columns labeled 82 and 119 in Figure 3A), but does not affect the two-hybrid interaction (see Figure 1). The inconsistency between clones 59 and 119 may be explained in a similar fashion.

In summary, the surface-located domain composed of K159-N161 and Q324 amino acid residues is a very convincing candidate for the long-sought PTS3 import signal identified for *S. cerevisiae* AOx. Thus, our data revealed the matching partner to the prospective PTS3-recognizing domain within the N-terminal region of Pex5p, which was discovered almost two decades ago. Remarkably, the data indicate that the PTS3 is not a linear sequence as PTS1 and PTS2 but is composed of amino acids that are distant from each other in the primary sequence but are in close proximity in the folded protein. Thus, in contrast to the linear PTS1 and PTS2, the PTS3 is a signal patch.

The data presented in this paper provide the basis for further studies on the import mechanisms of AOx and similar proteins from other species. One open question concerns the structure of analogous PTS3 signals within Cat2p, Fox2p and Cta1p, the peroxisomal import of which can be driven exclusively or partially by their interaction with the 250–270 amino acid region of Pex5p. Another intriguing finding provoking further studies is the possible involvement of FAD-binding in AOx interaction with its receptor and in peroxisomal import. Notably, the amino acid substitution within the FAD-binding fold of the *H. polymorpha* alcohol oxidase was also shown to abolish its peroxisomal import (Gunkel et al., 2004), indicative for cofactor binding being a prerequisite for peroxisomal protein import. Better characterization of this and other peroxisomal import mechanisms divergent from the canonical PTS1- and PTS2-dependent ones will contribute to our understanding of the evolution of the multi-purpose functions of these exciting organelles.

## Data Availability Statement

The datasets generated for this study are available on request to the corresponding author.

## Author Contributions

MS and RE: conceptualization and funding acquisition. BK, AC, JP, KK, RE, and MS: methodology. RE and MS: validation. MS: formal analysis, resources, data curation, visualization, supervision, and project administration. BK, AC, JP, KK, ŁR, ZF, WG, AS, and MS: investigation. ER and MS: writing – original draft, review, and editing.

## Conflict of Interest

The authors declare that the research was conducted in the absence of any commercial or financial relationships that could be construed as a potential conflict of interest. The handling Editor declared a past collaboration with one of the authors RE.

## Footnotes

1 www.yasara.org

## References

[B1] Chang J. Rachubinski R. A. (2019). Pex20p functions as the receptor for non-PTS1/non-PTS2 acyl-CoA oxidase import into peroxisomes of the yeast *Yarrowia lipolytica*. *Traffic* 20 504–515. 10.1111/tra.12652 31042004

[B2] Effelsberg D. Cruz-Zaragoza L. D. Tonillo J. Schliebs W. Erdmann R. (2015). Role of Pex21p for Piggyback import of Gpd1p and Pnc1p into Peroxisomes of *Saccharomyces cerevisiae*. *J. Biol. Chem.* 290 25333–25342. 10.1074/jbc.M115.653451 26276932 PMC4646183

[B3] Girzalsky W. Saffian D. Erdmann R. (2010). Peroxisomal protein translocation. *Biochim. Biophys. Acta* 1803 724–731. 10.1016/j.bbamcr.2010.01.002 20079383

[B4] Glover J. R. Andrews D. W. Rachubinski R. A. (1994). *Saccharomyces cerevisiae* peroxisomal thiolase is imported as a dimer. *Proc. Natl. Acad. Sci. U.S.A.* 91 10541–10545. 10.1073/pnas.91.22.10541 7937990 PMC45057

[B5] Guarente L. (1983). Yeast promoters and lacZ fusions designed to study expression of cloned genes in yeast. *Meth. Enzymol.* 101 181–191. 10.1016/0076-6879(83)01013-7 6310321

[B6] Gunkel K. van Dijk R. Veenhuis M. van der Klei I. J. (2004). Routing of *Hansenula polymorpha* alcohol oxidase: an alternative peroxisomal protein-sorting machinery. *Mol. Biol. Cell* 15 1347–1355. 10.1091/mbc.E03-04-0258 14699075 PMC363140

[B7] James P. Halladay J. Craig E. A. (1996). Genomic libraries and a host strain designed for highly efficient two-hybrid selection in yeast. *Genetics* 144 1425–1436. 8978031 10.1093/genetics/144.4.1425 PMC1207695

[B8] Kim S. Kim K.-J. (2018a). Crystal structure of Acyl-CoA oxidase 3 from *Yarrowia lipolytica* with specificity for short-chain Acyl-CoA. *J. Microbiol. Biotechnol.* 28 597–605. 10.4014/jmb.1711.11032 29429324

[B9] Kim S. Kim K.-J. (2018b). Structural insight into the substrate specificity of acyl-CoA oxidase1 from *Yarrowia lipolytica* for short-chain dicarboxylyl-CoAs. *Biochem. Biophys. Res. Commun.* 495 1628–1634. 10.1016/j.bbrc.2017.11.191 29198706

[B10] Klein A. T. J. van den Berg M. Bottger G. Tabak H. F. Distel B. (2002). *Saccharomyces cerevisiae* acyl-CoA oxidase follows a novel, non-PTS1, import pathway into peroxisomes that is dependent on Pex5p. *J. Biol. Chem.* 277 25011–25019. 10.1074/jbc.M203254200 11967269

[B11] Kragler F. Langeder A. Raupachova J. Binder M. Hartig A. (1993). Two independent peroxisomal targeting signals in catalase A of *Saccharomyces cerevisiae*. *J. Cell Biol.* 120 665–673. 10.1083/jcb.120.3.665 8425895 PMC2119545

[B12] Rymer Ł Kempiński B. Chełstowska A. Skoneczny M. (2018). The budding yeast Pex5p receptor directs Fox2p and Cta1p into peroxisomes via its N-terminal region near the FxxxW domain. *J. Cell. Sci.* 131:jcs216986. 10.1242/jcs.216986 30131444

[B13] Skoneczny M. Lazarow P. B. (1998). A novel, non-PTS1, peroxisomal import route dependent on the PTS1 receptor Pex5p. 38th American Society for Cell Biology Annual Meeting. *Mol. Biol. Cell* 9:348.

[B14] Small G. M. Szabo L. J. Lazarow P. B. (1988). Acyl-CoA oxidase contains two targeting sequences each of which can mediate protein import into peroxisomes. *EMBO J.* 7 1167–1173. 10.1002/j.1460-2075.1988.tb02927.x 3402435 PMC454452

[B15] Titorenko V. I. Nicaud J.-M. Wang H. Chan H. Rachubinski R. A. (2002). Acyl-CoA oxidase is imported as a heteropentameric, cofactor-containing complex into peroxisomes of *Yarrowia lipolytica*. *J. Cell Biol.* 156 481–494. 10.1083/jcb.200111075 11815635 PMC2173332

[B16] Walter T. Erdmann R. (2019). Current advances in protein import into Peroxisomes. *Protein J.* 38 351–362. 10.1007/s10930-019-09835-6 31054036

[B17] Yang X. Purdue P. E. Lazarow P. B. (2001). Eci1p uses a PTS1 to enter peroxisomes: either its own or that of a partner. *Dci1p. Eur. J. Cell Biol.* 80 126–138. 10.1078/0171-9335-00144 11302517

