# Supplementary material for: The Peroxisomal Targeting Signal 3 (PTS3) of the Budding Yeast Acyl-CoA Oxidase Is a Signal Patch
Source: Front Cell Dev Biol. 2020 Mar 27;8:198. doi: 10.3389/fcell.2020.00198 (PMC7135854; doi:10.3389/fcell.2020.00198)
Supplement: Supplementary file 1 [file Data_Sheet_1.docx]

***Supplementary Material***

**1 Materials and Methods, expanded version**

*Strains and media:* XL1-Blue (*recA1 endA1 gyrA96 thi-1 hsdR17 supE44 relA1 l* F´ *proAB lacIqZΔM15 Tn10 Tetr*) (Stratagene) bacterial strain was used for plasmid construction and propagation.

*Yeast strain*s: PJ69-4A, *MAT***a** *trp1-901 leu2-3,112 ura3-52 his3-200 gal4Δ gal80Δ LYS2::GAL1-HIS3 ade2-101::GAL2-ADE2 met2::GAL7-lacZ* (James et al., 1996), was used as a host for two-hybrid screens and tests. BYpex5Δ,pox1Δ, (*MAT***a** *his3Δ1 leu2Δ0 met15Δ0 ura3Δ0* *pox1::KanMX* *pex5::KanMX)* a double deletion derivative of BY4741 (Open Biosystems, Huntsville, AL, USA) was obtained by crossing of single deletion strains of opposite mating type and selecting double deletion segregant among the progeny. Plasmid-transformed bacteria were grown in solid and liquid LB media supplemented with 50μg per ml ampicillin at 37°C. Yeast strains were grown at 28°C either in YP media (1% yeast extract, 2% bactopeptone) or in synthetic complete (SC) selective media (0.67% Yeast Nitrogen Base and aminoacid mixture lacking the appropriate amino acids: histidine, leucine, tryptophan, uracil, adenine, or the combination thereof, as required to maintain the plasmids). 2% agar was added for solid media. For most applications 2% glucose was used as a carbon source. To induce peroxisome proliferation a mixture of 0.1% glucose, 2% ethanol, 0.1% oleic acid and 0.25% Tween 80 was used as a carbon source. Media ingredients were from Difco^TM^, Becton, Dickinson & Co, Sparks, MD, USA). All other reagents were of analytical purity grade.

Table S1. Plasmids that were used in this study

| Plasmid name | Reference |
| --- | --- |
| pGAD424 | (James et al., 1996) |
| pGBT9 | (James et al., 1996) |
| pT7Blue® | Novagen |
| pRS413 | (Sikorski and Hieter, 1989) |
| pGBT-AOxWT | (Skoneczny and Lazarow, 1998) |
| pGAD-Pex5^136-292^ | (Skoneczny and Lazarow, 1998) |
| pGAD-Pex5^136-292^I264K | this study |
| pRS-Pex5pWT | (Rymer et al., 2018) |
| pRS-Pex5pI264K | (Rymer et al., 2018) |
| pRS413-Pex5pWT | this study |
| pRS413-Pex5pIK | this study |
| AOxWT-GFP | (Rymer et al., 2018) |
| mRFP-SKL | (Rymer et al., 2018) |
| pGBT-AOx15 | this study |
| pGBT-AOx24 | this study |
| pGBT-AOx29 | this study |
| pGBT-AOx40 | this study |
| pGBT-AOx53 | this study |
| pGBT-AOx59 | this study |
| pGBT-AOx61 | this study |
| pGBT-AOx82 | this study |
| pGBT-AOx83 | this study |
| pGBT-AOx104 | this study |
| pGBT-AOx113 | this study |
| pGBT-AOx119 | this study |
| AOx15-GFP | this study |
| AOx24-GFP | this study |
| AOx29-GFP | this study |
| AOx40-GFP | this study |
| AOx53-GFP | this study |
| AOx59-GFP | this study |
| AOx61-GFP | this study |
| AOx82-GFP | this study |
| AOx83-GFP | this study |
| AOx113-GFP | this study |
| AOx119-GFP | this study |

*Plasmid construction:* To construct pGBT-AOx two-hybrid plasmid encoding AOx fused to Gal4p activating domain (AD), a 2695 bp *Ase*I fragment containing the *POX1* ORF beginning at codon 8 and ending 476bp downstream from the stop codon was cloned into the *Nde*I site of pT7Blue® vector (Novagen) to create the intermediate construct pT7-AOx. A clone with properly oriented insert was digested partially with *Eco*RI and completely with *Sal*I and the insert was cloned into *Eco*RI/*Sal*I-linearized pGBT9 vector. pGAD-Pex5p^136-292^ encoding Gal4p TF activating domain (AD) fused to short, 136-292 aminoacid fragment of Pex5p was the clone selected from the two hybrid library (James et al., 1996) in the screen for AOx-interacting polypeptides (Skoneczny and Lazarow, 1998) and contained a fragment of *PEX5* ORF between *Aci*I sites at 404 and 877 bp inserted into *Cla*I site of pGAD vector behind AD-encoding sequence. pGAD-Pex5^136-292^I264K plasmid encoding I264K-substituted fragment of Pex5p receptor fused to activating domain was obtained by PCR-driven site-directed mutagenesis of pGAD-Pex5p^136-292^ plasmid with I264K_up and I264K_lo primers. To subclone the mutated derivatives of AOxWT-GFP, NruI-BspEI fragments from mutated pGBT-AOx plasmids encompassing the mutated region were cloned into AOxWT-GFP digested with the same restriction enzymes. pRS413-Pex5pWT, pRS413-Pex5pIK were prepared by cloning the XhoI-XbaI fragments, containing the whole *PEX5* genes, from pRS-Pex5pWT or pRS-Pex5pI264K, respectively, into pRS413 vector (Sikorski and Hieter, 1989).

*Two-hybrid screen for amino acid substitutions within AOx polypeptide that affect its interaction with N-terminal region of Pex5p receptor. Construction of mutated POX1 libraries.* Four libraries were prepared to generate mutations in four parts of *POX1* gene: between the beginning of *POX1* ORF to Nru1 site at 303 bp, between NruI and ClaI site at 1519 bp, between ClaI and BspEI site at 1900 bp and between BspEI and the end of *POX1* ORF. Each fragment was PCR-amplified with the pair of appropriate primers (see Table S2) using GeneMorph II Random mutagenesis kit (Agilent, Santa Clara, CA, USA) according to manufacturer protocol to obtain on average single mutation per 1000 bp.

Table S2. Primers used to mutagenic amplification of fragments of *POX1* gene coding sequence.

| **Primer name** | **Description** | **Sequence** |
| --- | --- | --- |
| SacI_up_GR  NruI_lo_GR | Primers to amplify fragment I of *POX1* ORF | AGCATAGAATAAGTGCGACAT  ACAAGTGACAATCTCTTATCG |
| NruI_up_GR  ClaI_lo_GR | Primers to amplify fragment II of *POX1* ORF | TACGGACTATTACGATGCTA  ACCACTCTTTATTTCTCCAG |
| ClaI_up_GR  BspEI_lo_GR | Primers to amplify fragment III of *POX1* ORF | GTAAAGGCTATGACGACT  TGGCAAAAGAGCCAATAG |
| BspEI_up_GR  SalI_lo_GR | Primers to amplify fragment IV of *POX1* ORF | CAAAGGAATCTATGTGGAATG  CAGGAAAGAGTTACTCAAG |
| I264K_up  I264K_lo | Primers to introduce the point mutation into PEX5 gene fragment at codon 264 | CTTCTTCAGCGTCCTTGTGTTTGCTATCCCACACTTCTTGG  CCAAGAAGTGTGGGATAGCAAACACAAGGACGCTGAAGAAG |

The two hybrid libraries were made directly in yeast cells using gap repair method by transforming each of PCR products together with pGBT9-AOx two-hybrid plasmid linearized with the respective restriction enzyme pairs: SacI+NruI, NruI+ClaI, ClaI+BspEI or BspEI+SalI, into PJ69-4A strain previously transformed with pGAD-Pex5p^136-292^ plasmid. Transformant colonies grown on solid SC media lacking leucine and tryptophan were replica-plated on SC media lacking leucine, tryptophan, histidine and adenine. Colonies displaying affected growth on this medium were reanalyzed to confirm growth phenotypes on the medium without adenine. pGBT-AOx plasmid DNA from the prospective clones were isolated, amplified and transformed back to PJ69-4A/pGAD-Pex5p^136-292^ strain to finally confirm growth phenotypes. The inserts from selected clones encompassing *POX1* ORF were sequenced to identify the mutations.

*Quantification of two-hybrid interactions between AOx and N-terminal region of Pex5p*. pGBT-AOx plasmids bearing mutations within *POX1* ORF, selected in two-hybrid screen were transformed into PJ69-4A strain together with pGAD-Pex5p^136-292^ plasmid. In parallel, control transformations were done with empty pGAD424 plasmid and with pGBT-AOx bearing no mutations or with empty pGBT9 plasmid. Two-hybrid interaction displayed by transformant clones prepared in this manner was quantified in two ways. Clones pregrown overnight/to saturation in SC lacking leucine and tryptophan were serially diluted and spotted onto solid SC media lacking leucine, tryptophan, histidine and adenine and lacking leucine and tryptophan as a control of spotting uniformity. Plates were incubated for 2 days and their images were acquired by the flat-bed scanner.

Transformant clones were also inoculated into SC medium lacking leucine, tryptophan with 2% ethanol, 0.05% glucose as a carbon source and grown overnight. Yeast cells were collected by centrifugation, suspended in Z-buffer (60mM Na_2_HPO_4_, 40mM NaH_2_PO_4_, 10mM KCl, 1mM MgSO_4_, pH7.0), disrupted with glass beads in MiniBeadBeater-16 (BioSpec Products, Bartlesville, OK USA) and centrifuged in a microcentrifuge at max speed. 50-200μl of supernatants was taken for the β-galactosidase assay with O-Nitrophenyl-β-D-galactopyranoside (ONPG, Sigma Aldrich) as substrate, performed according to (Guarente, 1983). Protein concentration in the supernatants was measured according to (Lowry et al., 1951). Activity was expressed as nanomoles of ONPG hydrolyzed per minute of incubation time at 30°C per mg of protein. Assays were done in triplicates for three separate transformants of each two-hybrid plasmid combination.

*Modeling the structure of ScAOx*.

Yasara Structure v. 19.5.5 ([www.yasara.org](http://www.yasara.org)) protein modeling software was used to model the structure of *S. cerevisiae* AOx based on its homology to acyl-CoA oxidase proteins in other organisms, for which crystallographic structures are known.The structures of acyl-CoA oxidase of *Yarrowia lipolytica* (5YS9; 5Y9D), *Rattus norvegicus* (1IS2; 2DDH) and *Arabidopsis thaliana* (1W07) were automatically selected as the best templates according to the combination of the Blast E-value, sequence coverage, and structure quality. For each template, up to five alternate alignments with the target sequence were used, and up to 50 different conformations were tested for each modeled loop. The resulting models were evaluated according to structural quality (dihedral distribution, backbone and side-chain packing). The best-scored model was built on 5YS9 structure, however, the chimerical model, in which some parts were adopted from other highly-scored models (2DDH, 1W07) was scored even better (see Supplementary video 1 for the regions improved by this procedure). The final model contained the FAD molecule, location of which was taken directly from the template 5YS9 structure.

*In vivo import assay of AOx bearing amino acid substitutions identified in two-hybrid screen*. To transfer the mutations within the *POX1* ORF of pGBT-AOx two-hybrid plasmid the fragments between NruI and BspEI restriction sites were cloned into AOx-GFP plasmid (Rymer et al., 2018) digested with the same restriction enzymes. Plasmids were transformed into BYpex5Δ,pox1Δ yeast strain containing pRS413-Pex5WT or pRS413-Pex5IK plasmids and mRFP-SKL plasmid encoding the peroxisomal marker (Rymer et al., 2018). Transformant cells were pre-grown overnight at 28°C in SC selective medium without histidine, leucine and uracil with glucose as a carbon source, then inoculated into SC selective induction medium (see above) and grown at 28°C for 44 hours. 1 ml of culture was centrifuged, cells were washed twice with phosphate-buffered saline (PBS) and sedimented cells were resuspended in 50 μl of PBS. Cell images were acquired with Zeiss Axio Imager.M2 microscope with AxioCam MRc5 camera and AxioVision release 4.8 software under 1000x magnification with EC Plan-NEOFLUAR 100x objective, 38HE green filter and 63HE red filter for fluorescence and with Nomarski optics for bright-field imaging. Exposure times were 1500 ms for green fluorescence channel and 300 ms for red fluorescence channel and for bright-field. Three transformant clones were analyzed for each mutation and for the wild-type AOx-GFP fusion and for each clone at least 100 cells were categorized as displaying peroxisomal, mixed or cytosolic localization of AOx-GFP protein. The results for three independent clones expressing each of mutated AOx-GFP fusions or its variant with no mutations as an experimental control were averaged, standard deviation was calculated and the statistical significance was calculated with the Student’s t-test.

**References:**

Guarente, L. (1983). Yeast promoters and lacZ fusions designed to study expression of cloned genes in yeast. *Meth. Enzymol.* 101, 181–191. doi:10.1016/0076-6879(83)01013-7.

James, P., Halladay, J., and Craig, E. A. (1996). Genomic libraries and a host strain designed for highly efficient two-hybrid selection in yeast. *Genetics* 144, 1425–1436.

Lowry, O. H., Rosebrough, N. J., Farr, A. L., and Randall, R. J. (1951). Protein measurement with the Folin phenol reagent. *J. Biol. Chem.* 193, 265–275.

Rymer, Ł., Kempiński, B., Chełstowska, A., and Skoneczny, M. (2018). The budding yeast Pex5p receptor directs Fox2p and Cta1p into peroxisomes via its N-terminal region near the FxxxW domain. *J. Cell. Sci.* 131. doi:10.1242/jcs.216986.

Sikorski, R. S., and Hieter, P. (1989). A system of shuttle vectors and yeast host strains designed for efficient manipulation of DNA in Saccharomyces cerevisiae. *Genetics* 122, 19–27.

Skoneczny, M., and Lazarow, P. B. (1998). A novel, non-PTS1, peroxisomal import route dependent on the PTS1 receptor Pex5p. *Molecular Biology of the Cell* 9, 348A.

**2 Legend to Supplementary figure**

**Supplementary Figure 1**

Quantification of the *in vivo* import of AOx C-terminally tagged with GFP, bearing the amino acid substitutions identified in the two-hybrid screen. Individual columns on the graph are labeled with numbers referring to the original two-hybrid clones and are ordered as in Figure 1. Clone no. 104 was omitted because it had the same mutation as clone no. 24. For comparison, the data for the import of wild-type AOx-GFP either in the presence of wild-type (WT) or in the presence of I264K-substituted (+IK) Pex5p or in the absence of Pex5p (Δ) are shown. Plasmids encoding wild-type and mutated AOx-GFP proteins are described in Supplementary Data file. Cells were categorized as displaying peroxisomal (P), cytosolic (C) or mixed peroxisomal/cytosolic (M) localization of AOx-GFP and the number of cells is expressed as a percent of total number of cells counted. Error bars represent the standard deviation. Statistical significance was determined for peroxisomal and cytosolic localization data (upper and lower row of stars, respectively), relative to wild-type AOx-GFP (WT column) and was calculated with Student’s t-test: *** - P<0.005, ** - P<0.01, * - P<0.05, (*) - P<0.1. This figure is supplementary to Figure 3A in the main text.

**2 Legends to Supplementary videos**

**Supplementary video 1**

The model of *S. cerevisiae* AOx generated with Yasara Structure v. 19.5.5 software on the templates of known crystallographic structures of acyl-CoA oxidase of *Yarrowia lipolytica* (5YS9; 5Y9D), *Rattus norvegicus* (1IS2; 2DDH) and *Arabidopsis thaliana* (1W07). The majority of modeled structure is based on 5YS9 template (marked in blue or gray). Small fragments are modeled on 1IS2 (yellow), 5Y9D (gold) and 1W07 (green).

**Supplementary video 2**

The model of *S. cerevisiae* AOx generated with Yasara Structure v. 19.5.5 software. Amino acid residues crucial for AOx interaction with Pex5p and for its peroxisomal import are highlighted as in Figure 3.

**Supplementary video 3**

The enlargement of the region of *S. cerevisiae* AOx model shown in Supplementary video 2, generated with Yasara Structure v. 19.5.5 software. Amino acid residues crucial for AOx interaction with Pex5p and for its peroxisomal import are highlighted as in Figure 3 and Supplementary video 2.
