# Supplementary figures and images for: The Peroxisomal Targeting Signal 3 (PTS3) of the Budding Yeast Acyl-CoA Oxidase Is a Signal Patch
Source: Front Cell Dev Biol. 2020 Mar 27;8:198. doi: 10.3389/fcell.2020.00198 (PMC7135854; doi:10.3389/fcell.2020.00198)

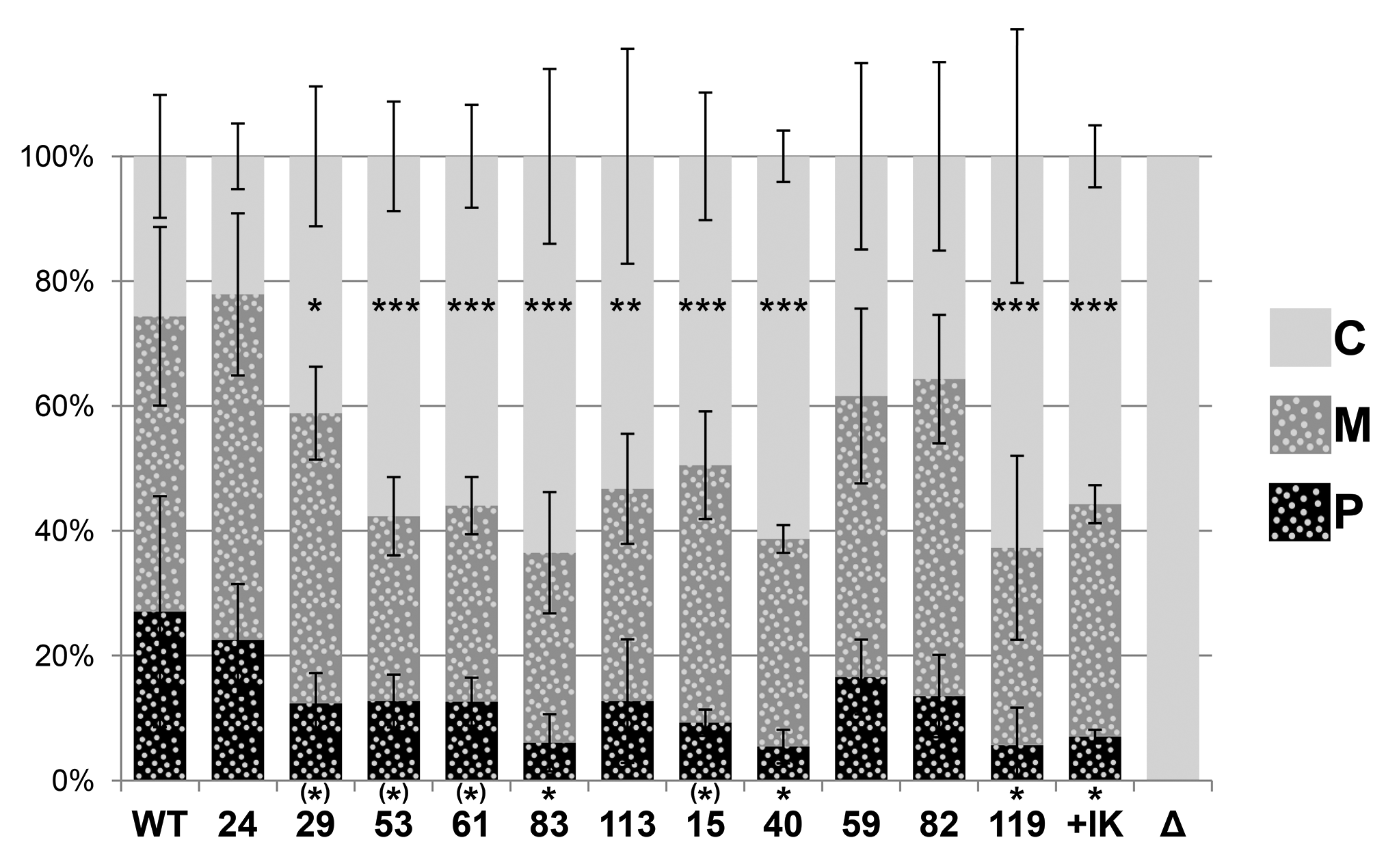

Supplement: Supplementary file 2 [file Image_1.TIF]
